# Time With Glucose Level in Target Range Among Children and Adolescents With Type 1 Diabetes After a Software Update to a Closed-Loop Glucose Control System

**DOI:** 10.1001/jamanetworkopen.2022.28669

**Published:** 2022-08-24

**Authors:** Marco Marigliano, Andrea E. Scaramuzza, Riccardo Bonfanti, Ivana Rabbone, Riccardo Schiaffini, Sonia Toni, Valentino Cherubini

**Affiliations:** 1 Pediatric Diabetes and Metabolic Disorders Unit, Regional Center for Pediatric Diabetes, University Hospital, Verona, Italy; 2 Pediatric Diabetes, Endocrinology and Nutrition, Division of Pediatrics, Azienda Socio-Sanitaria Territoriale Cremona, Ospedale Maggiore, Cremona, Italy; 3 Unità Operativa Pediatric Diabetes Research Institute, Ospedale San Raffaele, Milan, Italy; 4 Division of Pediatrics, Department of Health Sciences, University of Piemonte Orientale, Novara, Italy; 5 Diabetes Unit, Ospedale Pediatrico Bambino Gesù, Roma, Italy; 6 Pediatric Endocrinology and Diabetology Unit, Meyer Children’s Hospital, Firenze, Italy; 7 Pediatric Endocrinology and Diabetology Unit, Department of Women’s and Children’s Health, Azienda Ospedaliera Universitaria Ospedali Riuniti di Ancona, “G. Salesi” Hospital, Ancona, Italy

## Abstract

This cohort study analyzes changes to the time with glucose level in target range among children and adolescents with type 1 diabetes after a software update to a closed-loop glucose control system.

## Introduction

For people with type 1 diabetes, the percentage of time in range (TIR; 70-180 mg/dL [to convert to millimoles per liter, multiply by 0.0555]) is recognized as the most effective metric with glycated hemoglobin to assess glycemic control.^1^ Closed-loop control systems, such as the t:slim X2 insulin pump with Control-IQ technology (Tandem Diabetes Care Inc), have been reported to increase TIR by 9% among children and adolescents with type 1 diabetes,^2^ despite the difficulty in achieving glycemic targets in this age group.^3,4^ Education is also important to optimize glucose control when a new technology is adopted.^5,6^ We therefore analyzed immediate changes in TIR among a group of children and adolescents with type 1 diabetes switching from Tandem Basal-IQ technology to Control-IQ technology.

## Methods

In 2020, when an upgraded closed-loop system was introduced in Italy, a virtual educational camp (vEC) was organized for children and adolescents with type 1 diabetes.^5,6^ Nineteen Italian pediatric diabetes centers participated in this IRB-approved, prospective, multicenter clinical cohort study, which was approved by the Azienda Socio-Sanitaria Territoriale Cremona institutional review board. Patients’ parents provided written consent for participation. Patients aged 6 to 17 years who had used the previous closed-loop system for at least 3 months with carbohydrate counting and were available to test the upgraded closed-loop system and share their data on data-syncing software were eligible to be enrolled and actively participate in the vEC from November 6 to 8, 2020.^5^ Details of the vEC are reported elsewhere.^1^ In brief, using Zoom videoconferencing software, children and their parents participated in a series of activities for 3 days, for 6 hours each day, either exercising guided by personal trainers or informative sessions (eg, carbohydrate counting, fine-tuning upgraded closed-loop system) held by diabetes experts, dieticians, and psychologists. After this program, enrolled patients updated the closed-loop control system software from previous to upgraded closed-loop system. Differences in TIR 1 week (excluding the day of update) and 3 weeks before and after the updates were analyzed. Time in range values were summarized using median (IQR) values and compared with the Wilcoxon signed rank test. All *P* values were from 2-sided tests and results were deemed statistically significant at *P* < .05. Within space limitations, this report followed the Strengthening the Reporting of Observational Studies in Epidemiology (STROBE) reporting guideline for cohort studies.

## Results

The 43 participants enrolled were aged 7 to 16 years (median, 12 years; IQR, 9-13 years), of whom 23 (53.5%) were girls. The duration of diabetes ranged from 2 to 13 years (median, 6 years; IQR, 4-9 years). The median body mass index *z* score was −0.2 (IQR, −0.6 to 0.2), and 19 participants (44.2%) were prepubertal according to the Tanner classification.

After the closed-loop control system was updated, TIR significantly increased compared with the previous closed-loop system after the first week (median, 75% [IQR, 70%-82%] vs 64% [IQR, 54%-74%]; *P* < .001] and remained steady for the entire 3-week observation period at a median of 76% (IQR, 69%-82%) (Figure). Furthermore, there was lower interindividual variability with the upgraded closed-loop system, as shown by the reduced IQR. Other glucometrics are shown in the Table. There were no severe adverse events (severe hypoglycemia or diabetic ketoacidosis) during the observation period. Participants therefore had a median 11% (95% CI, 9%-16%) higher TIR than before using a closed-loop control system after 1 week and a median 12% (95% CI, 8%-17%) higher TIR after 3 weeks, approximately 8% higher than other clinical published data.^2^ There was no increase in time below the range, confirming system’s safety.

**Figure.**
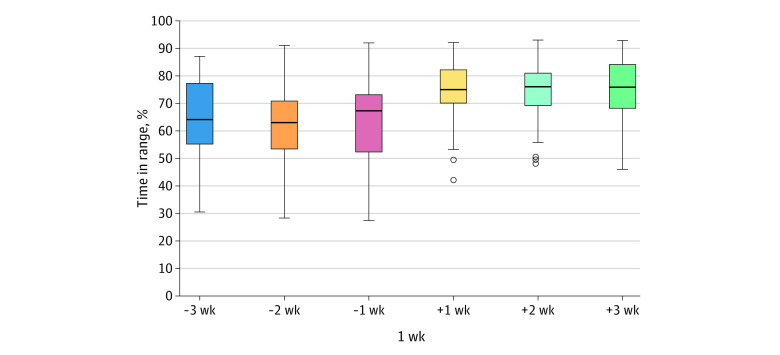
Early Time in Range Using the Upgraded Closed-Loop System Percentage of time in range 1, 2, and 3 weeks before and after updating to upgraded closed-loop system and participating in a virtual educational camp. The horizontal line in each box indicates the median, the boxes indicate the IQRs, the vertical lines indicate minimum and maximum data values (excluding the outliers), and the circles indicate the outliers.

**Table.** Overall CGM Outcomes Measured Before Updating to the Upgraded Closed-Loop System and 1 Week After the vEC Among 43 Children and Adolescents With Type 1 Diabetes

Outcome	Median (IQR)		Difference, median (95% CI)^a^	*P* value^b^
	Baseline	After 1 wk		
% Time in range, mg/dL				
<54	0 (0 to 1)	0 (0 to 1)	0 (−1 to 1)	.59
54-70	1 (1 to 3)	2 (1 to 3)	1 (−1 to 1)	.33
70-180	64 (54 to 74)	75 (70 to 81.5)	11 (9 to 16)	<.001
180-250	24 (20 to 28)	18 (12 to 24)	−6 (−8 to −4)	<.001
>250	9 (4.5 to 13.5)	4 (3 to 8.5)	−5 (−7 to −3)	<.001
CGM active, %	95 (93 to 97)	99 (97 to 99)	5 (−1 to 3)	.08
Mean BG, mg/dL	162 (149.5 to 171)	144 (139 to 161.3)	−18 (−20 to −9)	<.001
CV, %	36 (33 to 39)	35 (32 to 39)	−1 (−4 to 2)	.05
GMI, %	7.2 (6.8 to 7.4)	6.9 (6.6 to 7.3)	−0.3 (−0.5 to 0.1)	.004

Abbreviations: BG, blood glucose; CGM, continuous glucose monitoring; CV, coefficient of variation; GMI, glucose management indicator; vEC, virtual educational camp.

SI conversion factor: To convert glucose to millimoles per liter, multiply by 0.0555.

^a^ Differences are baseline vs after 1-week values.

^b^ Continuous glucose monitoring outcome *P* values refer to the Wilcoxon signed-rank test.

## Discussion

Our data show that it took only 1 week after switching to the closed-loop control system and attendance at a vEC for children and adolescents to attain a target TIR of 70 to 180 mg/dL at least 70% of the time.^1^ Time in range significantly increased after 1 week of using the upgraded closed-loop system, and this improvement was maintained over time.^5,6^ This study does, however, have some limitations. It is not possible to separate out the individual associations of education and technology with the TIR, although both factors are likely to be associated with the TIR. Moreover, the study participants had a fairly high (although below target) baseline TIR and were already using an advanced technology, which might affect study generalizability. It will be interesting to evaluate whether children and adolescents with lower baseline TIRs experience even greater benefit.

Nevertheless, our findings help to explore new strategies to engage youths in diabetes technology. The rapid improvement in glycemia provides an additional incentive to dedicate time to learning new systems. Closed-loop control systems, adequately supported by therapeutic education, might help to rapidly improve glycemic control and reach desired therapeutic goals for pediatric patients with type 1 diabetes.

## Notes

Supplement.
Nonauthor Collaborators
