# Supplementary material for: Time With Glucose Level in Target Range Among Children and Adolescents With Type 1 Diabetes After a Software Update to a Closed-Loop Glucose Control System
Source: JAMA Netw Open. 2022 Aug 24;5(8):e2228669. doi: 10.1001/jamanetworkopen.2022.28669 (PMC9403771; doi:10.1001/jamanetworkopen.2022.28669)
Supplement: Supplement. — Nonauthor Collaborators [file jamanetwopen-e2228669-s001.pdf]

Supplemental Online Content: Nonauthor Collaborators
*First name, last name, and suffix (if applicable) are required and will appear in PubMed.

*Group Name(s): Virtual Educational Camp (vEC) Study Group							
***First Name and Middle Initial(s)**	***Last Name**	***Suffix (eg, Jr, III)**	Academic Degrees	Institution	Location (city, state/province, country)	Role or Contribution, eg, chair, principal investigator	Group (if more than 1 Group listed in the byline) and/or Subgroup (eg, Steering Committee)
Federico	Abate Daga		CPT	Adapted Training and Performance Research Group, School of Exercise and Sport Sciences, University of Turin	Torino, Italy	Principal Investigator	
Marta	Bassi		MD	Pediatric Clinic, IRCCS G. Gaslini	Genova, Italy	Collaborator	
Maria Giulia	Berioli		MD	Department of Pediatrics, University of Perugia	Perugia, Italy	Principal Investigator	
Patrizia	Bruzzi		MD	Department of Medical and Surgical Sciences of the Mother, Children and Adults, University of Modena and Reggio Emilia	Modena, Italy	Collaborator	
Michela	Calandretti		CPT	Adapted Training and Performance Research Group, School of Exercise and Sport Sciences, University of Turin	Torino, Italy	Principal Investigator	
Chiara	Carducci		PsyD	Diabetes Unit, Pediatric Hospital Bambino Gesù	Roma, Italy	Principal Investigator	
Claudio	Cavalli		MD	Department of Pediatrics, ASST Cremona	Cremona, Italy	Collaborator	
Maurizio	Delvecchio		MD, PhD	Metabolic Diseases and Diabetology, Children’s Hospital Giovanni XXIII	Bari, Italy	Collaborator	
Rosaria	Gesuita		PhD	Centre of Epidemiology and Biostatistics, Polytechnic University of Marche	Ancona, Italy	Principal Investigator	
Sara	Giorda		RN	Department of Pediatrics, University of Torino	Torino, Italy	Principal Investigator	

Supplemental Online Content: Nonauthor Collaborators
*First name, last name, and suffix (if applicable) are required and will appear in PubMed.

*First Name and Middle Initial(s)	*Last Name	*Suffix (eg, Jr, III)	Academic Degrees	Institution	Location (city, state/province, country)	Role or Contribution, eg, chair, principal investigator	Group (if more than 1 Group listed in the byline) and/or Subgroup (eg, Steering Committee)
Dario	Iafusco		MD, PhD	Regional Center of Pediatric Diabetology, University of Campania “L. Vanvitelli”	Napoli, Italy	Principal Investigator	
Lorenzo	Lenzi		MD	Pediatric Endocrinology and Diabetology Unit, Meyer Children's Hospital	Firenze, Italy	Principal Investigator	
Fortunato	Lombardo		MD, PhD	Department of Human Pathology in adult and developmental age, University of Messina	Messina, Italy	Principal Investigator	
Donatella	Lo Presti		MD	Department of Pediatrics, University of Catania	Catania, Italy	Principal Investigator	
Claudio	Maffeis		MD, PhD	Pediatric Diabetes and Metabolic Disorders Unit, University of Verona	Verona, Italy	Principal Investigator	
Giulio	Maltoni		MD, PhD	Pediatric Unit, IRCCS, Azienda Ospedaliero-Universitaria	Bologna, Italy	Collaborator	
Chiara	Mameli		MD, PhD	Department of Pediatrics, Università di Milano, Buzzi Children’s Hospital	Milano, Italy	Principal Investigator	
Monica	Marino		RN	Department of Women’s and Children’s Health, G. Salesi Hospital	Ancona, Italy	Principal Investigator	
Barbara	Piccini		MD, PhD	Pediatric Endocrinology and Diabetology Unit, Meyer Children's Hospital	Firenze, Italy	Collaborator	
Nicola	Minuto		MD, PhD	Pediatric Clinic, IRCCS G. Gaslini	Genova, Italy	Principal Investigator	
Enza	Mozzillo		MD, PhD	Department of Translational Medical Science, Section of Pediatrics, Regional Center of Pediatric Diabetes, University of Naples Federico II	Napoli, Italy	Principal Investigator	

Supplemental Online Content: Nonauthor Collaborators
*First name, last name, and suffix (if applicable) are required and will appear in PubMed.

***First Name and Middle Initial(s)**	***Last Name**	***Suffix (eg, Jr, III)**	Academic Degrees	Institution	Location (city, state/province, country)	Role or Contribution, eg, chair, principal investigator	Group (if more than 1 Group listed in the byline) and/or Subgroup (eg, Steering Committee)
Elvira	Piccinno		MD	Metabolic Diseases and Diabetology, Children's Hospital Giovanni XXIII	Bari, Italy	Principal Investigator	
Ciro	Pignatiello		PsyD	Department of Health and Science, University of Piemonte Orientale	Novara, Italy	Principal Investigator	
Barbara	Predieri		MD, PhD	Department of Medical and Surgical Sciences of the Mother, Children and Adults, University of Modena and Reggio Emilia	Modena, Italy	Principal Investigator	
Francesca C	Redaelli		MD	Department of Pediatrics, Università di Milano, Buzzi Children's Hospital	Milano, Italy	Collaborator	
Maria Rossella	Ricciardi		MD	Department of Pediatrics and Microcythemia, ARNAS G. Brotzu	Cagliari, Italy	Collaborator	
Andrea	Rigamonti		MD	Department of Pediatrics, Diabetes Research Institute, San Raffaele Institute	Milano, Italy	Collaborator	
Carlo	Ripoli		MD, PhD	Department of Pediatrics and Microcythemia, ARNAS G. Brotzu	Cagliari, Italy	Principal Investigator	
Francesco M	Rosanio		MD	Department of Translational Medical Science, Section of Pediatrics, Regional Center of Pediatric Diabetes, University of Naples Federico II	Napoli, Italy	Collaborator	
Giuseppina	Salzano		MD, PhD	Department of Human Pathology in adult and developmental age, University of Messina	Messina, Italy	Collaborator	
Silvia	Savastio		MD	Department of Health and Science, University of Piemonte Orientale	Novara, Italy	Collaborator	

Supplemental Online Content: Nonauthor Collaborators
*First name, last name, and suffix (if applicable) are required and will appear in PubMed.

***First Name and Middle Initial(s)**	***Last Name**	***Suffix (eg, Jr, III)**	Academic Degrees	Institution	Location (city, state/province, country)	Role or Contribution, eg, chair, principal investigator	Group (if more than 1 Group listed in the byline) and/or Subgroup (eg, Steering Committee)
Valentina	Tiberi		MD	Department of Women's and Childre	Ancona, Italy	Collaborator	
Davie	Tinti		MD, PhD	Department of Pediatrics, University of Torino	Torino, Italy	Principal Investigator	
Michela	Trada		MD	Department of Pediatrics, University of Torino	Torino, Italy	Collaborator	
Sara	Zanetta		MD	Department of Health and Science, University of Piemonte Orientale	Novara, Italy	Collaborator	
Angela	Zanfardino		MD, PhD	Regional Center of Pediatric Diabetology, University of Campania "L. Vanvitelli"	Napoli, Italy	Principal Investigator	
Stefano	Zucchini		MD, PhD	Pediatric Unit, IRCCS, Azienda Ospedaliero-Universitaria	Bologna, Italy	Principal Investigator	
